# Addressing the sugar, salt, and fat issue the science of food way

**DOI:** 10.1038/s41538-018-0020-x

**Published:** 2018-07-16

**Authors:** Pingfan Rao, Raymond L. Rodriguez, Sharon P. Shoemaker

**Affiliations:** 1 Food and Nutrition Sciences Centre, hejiang Gongshang University, 310012 Hangzhou, China; 2 Department of Molecular and Cellular Biology, College of Biological Sciences, University of California, Davis, CA USA; 3 California Institute of Food and Agricultural Research, College of Agricultural and Environmental Sciences, University of California, Davis, CA USA

**Keywords:** Agriculture

Humans have had a long and beneficial relationship with salt, sugar, and fat that dates back to the origin of the species. Salt is essential for fluid balance, sugar provides the energy for physical and mental activity while fats of various types make up most of the mass of the brain. Over time, however, we discovered the seemingly magical properties of these three ingredients to transform smelly, stale, and near tasteless foods into sweet, savory, and delightfully flavorful nutrition. As a result of their ability to “flavorize” a vast array of foods, this trio of ingredients became a culinary treasure and used around the world to create those wonderful foods we have come to associate with important historical events, indispensable elements in religious rituals, feasts, festivals as well as those sweet memories from our youth.

By the mid 1900s, this trio of salt, sugar, fat took on a new psychosensory dimension when the processed food industry discovered that these ingredients could be formulated to produce a state of satiety, pleasure, and hedonia in those who consumed them. American market researcher and psychophysicist, Howard Moskowitz, termed this the “bliss point” or the point where the levels of saltiness, sweetness, and richness were perceived by the consumer as just right. When the processed food industry added a crunchy mouth feel to their bliss point formulations, a whole new generation of “craveable” foods was created.^1^ A vast array of craveable chips, dry sweetened cereals, candies, cookies, fried foods, and even spaghetti sauces became wildly popular among consumers, particularly children, and profits for processed food companies soared.

Of course, as interest and consumption of craveable foods surged, interest and consumption of more traditional, home cooked cuisine that included fresh fruits, vegetables, and whole grains began to wane. In terms of current sugar consumption, developed countries like the U.S. are consuming between 68 and 77 kg per year compared to the 1.8–2.7 kg consumed annually in the early 1700s.^2^ Interestingly, the introduction of sugar substitutes and government recommendations to lower the intake of sugar and salt have produced only slight reductions in recent years. It has been speculated by some in the fields of nutrition and biomedical research that these craveable foods can dysregulate the brain’s food reward system by increasing dopamine production, thus making them addictive.^1^

By 1999, the leaders of some of the largest processed-food companies in the U.S. met privately to discuss disturbing data that associated the consumption of craveable foods with an upturn in the rates of obesity, Type 2 diabetes, and cardiovascular disease. Equally disturbing was the finding that the rates of these diseases were higher in certain racial/ethnic populations suggesting strong genetic components in how people perceive the foods they eat (i.e., taste and smell) as well as how these foods interact with their physiology and metabolism. These findings spawned the new field of nutritional genomics that provided evidence for diet × genome interactions and genetic variations called single nucleotide polymorphism (SNPs). Some of these SNPs explained why certain individuals are “super tasters” for sugar while others prefer umami flavors. Nutritional genomics also helped explain why some people gain weight while other lose weight on the same isocaloric diet.

Today, craveable foods and soft drinks are sold and consumed worldwide and their negative impact on global health is being noticed in terms of increased rates of obesity and its comorbidities, diabetes, and cardiovascular disease.^3^ In some instances, food shortage and nutritional deficiencies in the developing world are being replaced with processed, bliss point-formulated, craveable foods resulting in increasing rate of “skinny diabetes” (Type 2 diabetes without obesity). As a consequence, the treasured trio of salt, sugar, and fat have become the subjects of much criticism by the public and extensive oversight by government agencies. For example, 11 countries have national taxes on sugar while there are nine cities in the U.S. with sugar taxes.

Is government regulation, taxation, and the demonization of salt, sugar, and fat the only way to curb the overconsumption and dependency on craveable foods? We, think not. We believe that the full force of food science and technology is yet to be applied to this challenging problem. For example, by emphasizing the use of highly refined ingredients in our processed foods and beverages, have we robbed them of their full and natural hedonic properties? Could the use of raw sugars and sea salt, for instance, help achieve bliss points at lower concentrations of these ingredients? Is there a bliss point equivalent for mixtures of fruits, vegetable, whole grains, fresh or processed? Are some bliss point-formulated foods more detrimental for one subpopulations than another? If so, can processed foods be re-formulated to achieve satiety and food reward based on racial/ethnic and socio-cultural factors instead of taking a “one size fits all” approach. Lastly, can consumers be sensorially trained to prefer lower bliss point foods without the loss of their hedonic properties?

These are just some of the exciting challenges facing food science and technology today. Addressing salt, sugar, and fat issue the “science of food” way is sure to produce the products and responses that will deliver those benefits of health and longevity that we all desire.

## Data availability

All relevant data are available from the authors upon request.
